# Cortical Modulation After Two Different Repetitive Transcranial Magnetic Stimulation Protocols in Similar Ischemic Stroke Patients

**DOI:** 10.21315/mjms2018.25.2.12

**Published:** 2018-04-27

**Authors:** Muhammad Hafiz Hanafi, Nur Karyatee Kassim, Al Hafiz Ibrahim, Munirah Mohd Adnan, Wan Muhamad Amir W Ahmad, Zamzuri Idris, Lydia Abdul Latif

**Affiliations:** 1 School of Medical Sciences, Universiti Sains Malaysia, 16150 Kubang Kerian, Kelantan, Malaysia; 2 Center for Neuroscience Services and Research, Universiti Sains Malaysia, 16150 Kubang Kerian, Kelantan, Malaysia; 3 School of Dental Sciences, Universiti Sains Malaysia, 16150 Kubang Kerian, Kelantan, Malaysia; 4 Department of Rehabilitation Medicine 2, Faculty of Medicine, University of Malaya, 50603 Kuala Lumpur, Malaysia

**Keywords:** stroke, motor evoked potential, repetitive transcranial magnetic stimulation

## Abstract

**Background:**

Stroke is one of the leading causes of mortality and morbidity in Malaysia. Repetitive transcranial magnetic stimulation (rTMS) is one of the new non-invasive modality to enhance the motor recovery in stroke patients.

**Objectives:**

This pilot study compared the motor evoked potential (MEP) changes using different settings of rTMS in the post-ischemic stroke patient. The goal of the study is to identify effect sizes for a further trial and evaluate safety aspects.

**Methods:**

Eight post-stroke patients with upper limb hemiparesis for at least six months duration were studied in a tertiary hospital in Northeast Malaysia. Quasi experimental design was applied and the participants were randomised into two groups using software generated random numbers. One of the two settings: i) inhibitory setting, or ii) facilitatory setting have been applied randomly during the first meeting. The motor evoked potential (MEP) were recorded before and after application of the rTMS setting. A week later, a similar procedure will be repeated but using different setting than the first intervention. Each patient will serve as their own control. Repeated measures ANOVA test was applied to determine the effect sizes for both intervention through the options of partial eta-squared (η^2^_p_).

**Result:**

The study observed large effect sizes (η^2^_p_ > 0.14) for both rTMS settings in the lesion and non-lesion sides. For safety aspects, no minor or major side effects associated with the rTMS was reported by the participants.

**Conclusions:**

The partial eta square of MEP value for both rTMS settings (fascilitatory and inhibitory) in both lesion and non-lesion sides represents large effect sizes. We recommend further trial to increase number of sample in order to study the effectiveness of both settings in ischemic stroke patient. Our preliminary data showed both settings may improve the MEP of the upper extremity in the ischemic stroke patient. No significant improvement noted when comparing both settings.

## Introduction

Stroke is a leading cause of death in Malaysia according to a 2013 report from the Malaysia Ministry of Health (1). This statistic showed that 25.10% of all deaths in Malaysia in 2012 were caused by stroke and cardiovascular diseases.

Standard stroke rehabilitation intervention usually enhances motor function recovery moderately rather than markedly (2). Cortes et al. (3) suggested that a more complete understanding of biological recovery is essential to improve rehabilitation interventions and boost function. Thus, many modalities have been added to standard stroke rehabilitation to significantly induce neuroplasticity that can translate into favourable motor gains (4).

Non-invasive brain stimulation is a collective term used when the brain region is modulated either for diagnostic or therapeutic purposes, or both. The two commonly used devices for non-invasive brain stimulation that induce neuromodulation in stroke are transcranial direct current stimulation (tDCS) and transcranial magnetic stimulation (TMS) (5). Both TMS and tDCS have been useful supportive tools that augment motor recovery in stroke (4).

TMS was first introduced in 1985 by Anthony Barker and his colleagues in England (6). This non-invasive intervention was preferred as it was painless and had been shown to induce changes in cortical excitability and functional improvement (7).

A motor evoked potential (MEP) is an electrical potential difference that is recorded using bipolar surface electromyography over the targeted muscle after stimulation is given using repetitive TMS (rTMS). Most commonly, distal intrinsic hand muscles are chosen due to their larger cortical representation in the M1 primary motor cortex area and have a lower motor threshold (8). The MEP is thought to give crucial knowledge on the physiological integrity of the primary motor cortex through the corticospinal tract and ending at the intended muscle (3). In summary, a gain in MEP from its initial value means that corticomotor excitability has increased and a reduction in MEP means that corticomotor excitability has decreased. But researchers need to bear in mind that MEP is a net value of various interactions of stimulation and inhibition in the corticospinal tract. Thus, a decrease in MEP can either be due to an increase in inhibitory activities in the tract or to a decrease in excitatory activities, or both (9).

MEP can be used as a quantitative physiological measurement for both diagnostic and therapeutic purposes in the stroke population. For diagnostic purposes, many studies (10, 11) have shown that the presence of MEP in the early phase of a stroke is a good indication of motor recovery in a stroke patient, and is thus a valuable marker of prognostication of stroke recovery (10). Another author (12) has suggested that MEP is an important sensitive investigation marker to evaluate motor recovery quantitatively in stroke. In one of the largest studies examining MEPs in stroke patients by Heald et al. (13) (118 patients were included in this study which followed the progression of stroke survivors for up to 12 months), they concluded that the presence of MEPs in post-stroke patients was associated with better survival and functional recovery. Likewise, the absence of MEPs indicates poor recovery and a higher mortality rate. Therapeutically, a systematic review of the effectiveness of rTMS in stroke populations found encouraging evidence that rTMS can help improve short-term upper extremity function and reduce mortality in chronic stroke patients (4). The systematic review did not suggest which rTMS setting (facilitatory or inhibitory) would be of more benefit to stroke patients.

Suzanne and Cathy (14) mentioned that motor cortex excitability is symmetrical between both brain hemispheres in healthy adults and that the amount of inhibition from one area of the primary motor cortex (the M1 area) to the other side of the primary motor cortex area is similar. Following a stroke, activity in this M1 area will be reduced in the lesion site and increased on the contralateral M1 area. This increase of activity in the contralesional site can increase the transcallosal inhibition signal given to the lesion site which may then impair the stroke recovery process in the lesioned hemisphere (15).

The literature review showed that inhibitory rTMS of the brain’s primary motor cortex (M1 area) on the non-lesional hemisphere of stroke patients will decrease excitability and hence reduce the transcallosal inhibition to the lesion side of the motor cortex (16). This inhibition can improve the recovery process of the lesion side of the brain by suppressing the inhibition signal from the non-lesion side. Alternately, facilitatory rTMS to the affected hemisphere could increase brain activity and thus, modulate neuroplasticity and motor recovery.

Repeated stimulation using TMS with one single intensity applied to a single brain area will disrupt cortical function. The disruption of the higher brain function is in direct proportion to the amplitude and frequency given during the therapy. If the stimulation is too low (less than 5 Hz), inhibition will be dominant, while if stimulation is higher (5 Hz–20 Hz), facilitation will be more prominent (17–20). A few human studies using a combination of rTMS and functional magnetic resonance imaging or positron emission tomography scans have shown evidence of a reduction in cerebral blood flow (CBF) and cerebral metabolism at 1 Hz stimulation, and an increase of CBF and metabolism at 10 Hz–20 Hz stimulation (19, 20).

In this study, we explored the improvement that MEPs can bring to ischaemic stroke patients. We changed the frequency and amplitude of rTMS in these patients, using both facilitation to the injured brain hemisphere, and inhibition of the contralesional hemisphere at different time frames. We then compared modalities to determine which one gave a better result. Patients did not experience any pain during the procedures, and pain reduction methods were not required. In addition, hospital stays were not needed during the treatment.

If successful, this rTMS study will become our guide in dealing with rTMS therapy in stroke patients. As the MEP has been shown to be directly proportionate with stroke recovery prognosis, rTMS can be introduced as a non-pharmacological alternative. This study can also serve as a pilot study to identify effect sizes for further trials and evaluate safety aspects.

## Methods

This study has a quasi-experimental study design to evaluate the effect sizes and safety aspects of the facilitatory and inhibitory settings on lesion and non-lesion sides. We applied a single blind experimental procedure to this study.

### Subjects

The study was approved by the medical ethics committee of the University Malaya Medical Centre. All patients completed and signed the appropriate consent forms prior to their tests. Inclusion criteria for participation in this study included a stabilised ischaemic stroke at least six months prior, a demonstrated minimum of at least 10° of active extension of the paretic index finger (the metacarpophalangeal joint) and no participation in other ongoing stroke research. Exclusion criteria included patients who were medically unstable, comorbidities in the upper or lower limbs, and problems associated with an inability to sit still for at least 30 min (during preparation and stimulation periods). Patients with pacemakers, spinal or bladder stimulators, previous skull openings or trauma, a history of epilepsy, or presence of foreign metallic bodies were also excluded from this study.

### Study Design

Safety was assessed based on the “Screening questionnaire before rTMS,” prepared by the International Federation of Clinical Neurophysiology, for the safety and ethical guidelines in the use of TMS in clinical practice and research (21).

**Figure 1:**
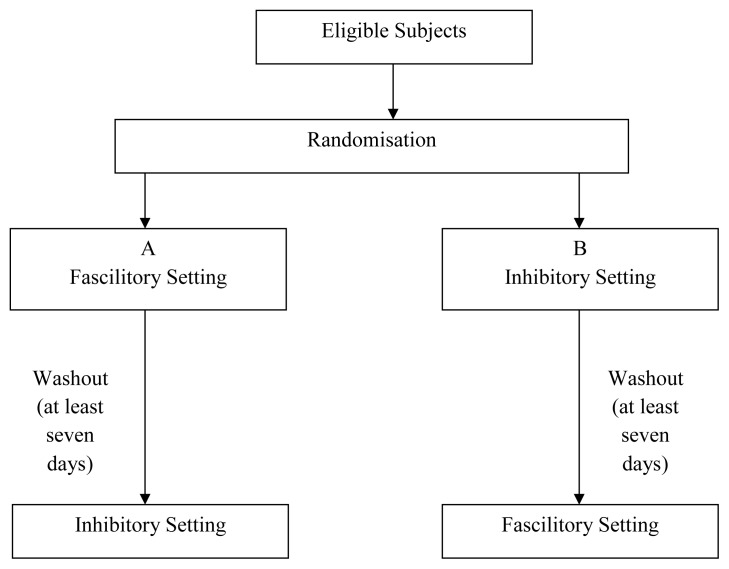
Diagram of participants flow

At the first visit, the suitability of the patients for the study was screened using the inclusion and exclusion criteria mentioned above. Subjects were randomised using IBM SPSS Statistics software version 22. The patients were divided into two groups of either receiving the facilitatory setting (high frequency) first then the inhibitory setting (low frequency), or the inhibitory setting first before the facilitatory setting. All subjects acted as their own control, and the interval between each stimulation session was at least seven days (the wash out period) to prevent any priming or carry-over effects. Participant flow in the study is summarised in Figure 1.

### Assessment of Cortical Excitability

Measurements with rTMS were done using a double 70 mm cooled coil system attached to an rTMS machine (Magstim Rapid^2^). The cooled coil in the double 70 mm configuration can be run for extended periods of time without overheating thus removing the need to replace coils during stimulation protocols. Excitability of both lesion and non-lesion cortical sides were measured using TMS.

The optimal site of stimulation (motor hot spot) on the skull was defined as the location where the largest recorded MEP occurred in the surface electromyography of the first dorsal interosseous (FDI) muscle of the unaffected upper limb. From there, the resting motor threshold (RMT) was established, defined as the lowest intensity of stimulation that could produce an MEP > 50 μV peak-to-peak amplitude of the muscle five times out of ten tries, with the patient at rest. The process was repeated ten times, and the mean of the 10 MEP amplitude readings was taken as the mean MEP for the FDI on the non-paretic side.

The same RMT intensity was then applied to the lesional side, and the mean MEP was recorded. An absent MEP was defined as failing to get an MEP > 50 μV peak-to-peak amplitude of the muscle after ten tries with the patient at rest.

Domains of interest in this study are the changes in physiological neuronal excitability (as recorded by the MEP readings) in both the paretic and non-paretic hand’s FDI muscle. For each subject, the mean MEP from 10 MEP recordings was calculated for both sides before each rTMS procedure.

### rTMS Protocols

One of the following two protocols was randomly applied alternately at a minimum of one week intervals (following current safety recommendations): i) an inhibitory application to the contralesional area comprising a series of focal 1 Hz rTMS to the non-lesional hemisphere over the M1 primary motor cortex at 90% RMT intensity for 20 min [1 Hz × 60 s × 20 min = 1200 pulses] (22), ii) a facilitatory application to the ipsilesional area comprising a series of 10 Hz rTMS at 80% RMT intensity to the lesional hemisphere M1 primary motor cortex with 50 pulses per 5 s train for 20 trains with a 25 s intertrain interval. [10 Hz × 5 s × 20 trains = 1000 pulses] (23).

After each protocol, the mean MEP from 10 MEP recordings was recalculated using the same method as above and any new changes in amplitude were recorded. There was a minimum seven-day resting period between both protocols to prevent the priming effect, thus allowing each patient to act as their own control.

### Statistical Analysis

Data was analysed using SPSS version 22. Mean and standard deviations (SDs) were calculated for continuous variables, and frequency and percentages for categorical variables. A repeated measures analysis of variance (ANOVA) was used to analyse the MEPs to compare the mean differences within each setting of rTMS (facilitatory and inhibitory). Effect sizes for both interventions were checked based on a partial eta-squared (η^2^_p_) while running the repeated measures ANOVA. All the data in this study were assessed using box plots and the Shapiro-Wilk test to ensure that there were no outliers, and that the data was normally distributed for each group. The significance value was set to 0.01.

## Results

Eight participants who complied with the inclusion and exclusion criteria and who consented to the study were included in our research. The rTMS sessions were conducted by the neurological rehabilitation team at the Medical Faculty of the University of Malaya. The clinical characteristics of the participants are shown in Table 1. The mean (SD) age of the study sample was 52.38 (SD = 7.29) years, and the mean (SD) period from the onset of the cerebrovascular accident was 21.50 (SD = 16.09) months. The eight participants had experienced two cortical strokes and six subcortical strokes, respectively. From this group, five subjects were Malay and the remaining three were Indian. There were seven males and only one female participant. Six of the eight patients had the lesion on their dominant brain hemisphere. There were no significant differences in baseline characteristics (age, infarct site, duration after stroke, and side of the lesion).

The use of both facilitatory and inhibitory rTMS protocols was successfully done without any unwanted detrimental side effects in all subjects and all MEP recordings were successfully accomplished. One of the patients was found to have no MEP reading (MEP reading below 50 μV) before the facilitatory setting of rTMS but later exhibited an MEP reading after the rTMS procedure. A similar result could not be reproduced using the inhibitory rTMS setting on the same subject. Another patient withdrew from the study after one session due to personal reasons.

### Cortical Excitability

Application of facilitatory stimulation to the lesion hemisphere of the brain and inhibitory stimulation to the non-lesion hemisphere were aimed at increasing the MEPs of the lesion side. We found that the MEP of the pre-facilitatory lesion side was indeed lower than that of the post-facilitatory lesion side with a mean (SD) of 56.20 (SD = 2.63) μV and 59.77 (SD = 5.28) μV, respectively. A similar increasing pattern of mean (SD) MEP changes was seen in the pre-inhibitory lesion side, 55.07 (SD = 5.80) μV, compared to the post-inhibitory lesion side, 59.27 (SD = 6.22) μV (Table 2).

The partial eta-squared (η^2^_p_) for the MEPs of the facilitatory (η^2^_p_ = 0.512) and inhibitory settings (η^2^_p_ = 0.269) of TMS in the lesion side represent large effect sizes (Table 3).

When the same MEP measurements were applied to the lesion side, we expected that the MEPs of the non-lesion side would reduce after both interventions. As expected, we found that the mean (SD) of the pre-facilitatory non-lesion side MEPs were higher at 282.15 (SD = 171.40) μV compared to post-facilitatory at 217.63 (SD = 84.21) μV. A similar decreasing pattern of MEP mean (SD) values was seen in the pre-inhibitory non-lesion side, 259.90 (SD = 128.60) μV when compared to the post-inhibitory non-lesion side, 156.44 (SD = 21.61) μV (Table 2).

The partial eta-squared for the MEPs of the facilitatory (η^2^_p_ = 0.166) and inhibitory settings (η^2^_p_ = 0.479) of TMS in the non-lesion side also represents large effect sizes, similar to the lesion side (Table 4).

### Safety Aspects

The median duration of assessment for adverse effects was 48 h after completing each intervention. This study relied on spontaneous reports by participants and direct questioning by the investigators. Minor adverse effects could include hearing problems, headaches, or palpitations. Major adverse effects could include seizures, hypomania, syncope, or burns from the coil placement. Fortunately, none of the participants reported any of these minor or major adverse side effects in this study.

## Discussion

Cortical stimulation in stroke patients can be achieved by both invasive and non-invasive methods (24). Both methods have shown promising results in improving motor recovery in stroke populations. The non-invasive method is preferred as a safe and painless examination of cortical and corticospinal physiology (25). According to a systematic review by Norine et al. (4), there is conflicting evidence that rTMS enhances upper extremity function and mobility in stroke patients. Instead, they found strong evidence that tDCS improved hand function in the chronic stages of stroke, with an anodal tDCS setting being more effective than a cathodal setting for stroke patients.

Conventional rTMS is thought to be less accurate in targeting the primary motor cortex. This hypothesis is supported by a 2011 study that a neuronavigation system for rTMS produces better physiological and behavioural effects than conventional rTMS (26). A neuronavigation rTMS system uses real-time visualisation and feedback of the coil position to improve targeting and stimulus delivery with better precision (27). However, we found that most of the rTMS studies from our literature review were done using conventional rTMS.

A 2013 study by Simis et al. (5) concluded that anodal tDCS and facilitatory rTMS induce different changes in cortical plasticity. They showed that both techniques, when applied to the lesional side, produced comparable motor improvement in the stroke population. But the two techniques induced opposing results in cortical excitability. High-frequency rTMS improved cortical excitability, whereas 20 min of tDCS induced the opposite effect. The researchers hypothesised that the paradoxical finding in the tDCS protocol may be due to homeostatic principles.

Another controversy is that low-frequency rTMS could lead to poor hand function on the non-paretic side because of a decrease in the excitability of the non-lesional area (24, 28). However, a recent meta-analysis in May 2014 contradicted this theory as they found no side effects in the healthy hand after rTMS (29). But the meta-analysis did note an increased risk of seizure in the facilitatory setting of 20 Hz–25 Hz when set to 120%–130% of resting motor potential. According to Rossi et al. (20), this risk of developing seizures from rTMS has been minimalised since the introduction of the rTMS guidelines, safety limits and safety questionnaire.

We conducted single sessions of rTMS to prevent carry-over effects and differentiate MEP changes between rTMS settings. Takeuchi et al. (29) showed that the effect of single session rTMS could last up to one week. For this reason, we chose a minimum interval of one week between each rTMS procedure to prevent any priming effect. Priming is an implicit memory of the brain in which an exposure to one stimulus influences a response to another stimulus.

Our finding suggests that although the MEPs on the lesion side showed improvement after both facilitatory and inhibitory applications, the gain was not enough to be statistically significant. An inverse proportion pattern of MEPs on the non-lesion side after both applications was also noted and, as in the previous group, was without significant statistical effect.

Many studies use both facilitatory and inhibitory settings of rTMS and show promising results in recovering upper extremity motor function in patients who had suffered acute strokes (9), but no study has compared the effectiveness between settings. This study was designed to analyse any statistically significant changes in MEP after subjects had undergone both settings of rTMS. As all subjects acted as their own controls, changes in MEP within the same subject could be calculated for each rTMS setting.

Kim et al. (30) successfully showed that a single session facilitatory rTMS to the affected hemisphere could enhance the MEPs of that side with a larger improvement in MEP amplitude as compared to sham rTMS sessions. Our study reports a similar finding. Similarly, Takeuchi et al. (29) used the inhibitory setting of rTMS to the non-affected side and noted improvement in the MEPs on the lesion side immediately with the effect being sustained for one week. Our study has reproduced their effect of the inhibitory rTMS setting improving the lesion side MEPs. The latest study by Thanakamchokchai et al. (31) has further supported the effectiveness of low-frequency rTMS and task-specific training in improving the motor recovery of the paretic limb.

In a clinical setting, this study shows that there is no statistically significant difference in using either a facilitatory rTMS setting on the affected side or an inhibitory rTMS setting on the non-affected side to improve the MEPs in ischaemic stroke patients. Physicians can use their clinical judgement to choose the appropriate rTMS setting that will be least harmful and most beneficial to their patients (i.e., patients with high seizure risk would receive an inhibitory setting). Such an approach would not risk patients receiving suboptimal rTMS treatments due to their comorbidities.

One interesting finding in this study concerned the patient who initially had no MEP reading on the lesion side. We recorded the emergence of an MEP reading after giving our patient facilitatory rTMS to the lesion side. However, there was no emerging MEP reading for that patient after receiving inhibitory rTMS to the non-affected side. Escudero et al. (10) had a similar finding. Seven of their 11 patients who were without MEP readings before the facilitatory rTMS to the affected sides, then manifested post-facilitatory MEP readings. They also found better clinical recovery (using Barthel index scores and handgrip strength) for those patients who exhibited MEP readings after facilitatory rTMS than those patients who had no MEPs either before or after the procedure. Future research should be performed to investigate this finding.

The study observed large effect sizes (η^2^_p_ > 0.14) for rTMS on both the lesion and non-lesion sides. This might be due to the small number of study participants. TMS is still a new rehabilitation technology in Malaysia, with a limited number of centres using it for either diagnostic or therapeutic purposes, or both. One of the participants in this study opted to withdraw after the first session due to his (and his family’s) unfamiliarity with this technology and fear of unwarranted side effects.

In conclusion, both low-frequency rTMS applied to the non-lesional hemisphere and high-frequency rTMS applied to the lesional hemisphere can improve the MEPs of upper extremity motor function in ischaemic stroke patients. There was no significant difference in the improvement between the two procedures. The partial eta-squared of MEP values for both rTMS settings (facilitatory and inhibitory) in both lesion and non-lesion sides represented large effect sizes.

## Figures and Tables

**Table 1:** Clinical and demographic data

Patient	Patient’s age (year)	Infarct Site	Duration After Stroke (months)	Patient’s Race	Patient’s Gender	Side of brain lesion
1	49	subcortical	32	Malay	male	Dominant, left
2	46	subcortical	31	Malay	male	Dominant, left
3	57	cortical	12	Indian	male	Dominant, left
4	52	subcortical	53	Indian	male	Non dominant, right
5	39	cortical	7	Indian	male	Dominant, left
6	58	subcortical	9	Malay	female	Dominant, left
7	59	subcortical	9	Malay	male	Dominant, left
8	59	subcortical	19	Malay	male	Non dominant, right

**Table 2:** Motor Evoked Potentials (in μV) in the lesion and non-lesion side pre- and post-facilitatory and inhibitory setting of rTMS

Setting	*n*	Mean	Std. Deviation
Pre-fascilitatory Lesion side	7	56.20	2.631
Post-fascilitatory Lesion side	7	59.77	5.284
Pre-inhibitory Lesion side	6	55.07	5.803
Post-inhibitory Lesion side	6	59.27	6.215
Pre-fascilitatory Non Lesion side	8	282.37	171.400
Post-fascilitatory Non Lesion side	8	217.63	84.213
Pre-inhibitory Non Lesion side	7	259.90	128.603
Post-inhibitory Non Lesion side	7	156.44	21.610

**Table 3:** Comparison of MEP (in μV) within each setting of rTMS (fascilitatory and inhibitory) in the lesion side^a^

Stimulation	Mean Diff (95% CI)	*F* stat (df)	*P*-value	Partial Eta Squared
Fascilitatory	3.57 (−2.57, 9.71)	6.298 (1)	0.046	0.512
Inhibitory	4.20 (−10.59, 18.99)	1.84 (1)	0.233	0.269

a Repeated Measures ANOVA at significance level of *P*-value < 0.01

**Table 4:** Comparison of MEP (in μV) within each setting of rTMS (fascilitatory and inhibitory) in the non-lesion side

Stimulation	Mean Diff (95% CI)	*F* stat (df)	*P*-value	Partial Eta Squared
Fascilitatory	−64.74 (−285.38, 155.90)	1.398 (1)	0.276	0.166
Inhibitory	−103.46 (−293.44, 86.52)	5.52 (1)	0.057	0.479

a Repeated Measure ANOVA at significance level of *P*-value < 0.01
